# Plasma Soluble Fibrin Is Useful for the Diagnosis of Thrombotic Diseases

**DOI:** 10.3390/jcm12072597

**Published:** 2023-03-30

**Authors:** Minoru Ezaki, Hideo Wada, Yuhuko Ichikawa, Nozomi Ikeda, Katsuya Shiraki, Akitaka Yamamoto, Isao Moritani, Motomu Shimaoka, Hideto Shimpo

**Affiliations:** 1 Department of Central Laboratory, Mie Prefectural General Medical Center, Yokkaichi 510-0885, Japan; 2 Department of General and Laboratory Medicine, Mie Prefectural General Medical Center, Yokkaichi 510-0885, Japan; wadahide@clin.medic.mie-u.ac.jp; 3 Department of Emergency and Critical Care Center, Mie Prefectural General Medical Center, Yokkaichi 510-0885, Japan; 4 Department of Gastroenterology, Mie Prefectural General Medical Center, Yokkaichi 510-0885, Japan; 5 Department of Molecular Pathobiology and Cell Adhesion Biology, Mie University Graduate School of Medicine, Tsu 514-8507, Japan; 6 Mie Prefectural General Medical Center, Yokkaichi 510-0885, Japan

**Keywords:** soluble fibrin (SF), D-dimer, FDP, thrombosis, DIC

## Abstract

Background: Soluble fibrin (SF) is a form of fibrinogen that is activated by thrombin and is considered to be useful for the diagnosis of the prethrombotic state or thrombosis. Methods: Plasma levels of fibrin-related markers (FRMs), such as SF, D-dimer, fibrinogen, and fibrin degradation prioduct (FDP) levels in critically ill patients, were examined for the diagnosis of disseminated intravascular coagulation (DIC), venous thromboembolism (VTE), peripheral arterial thromboembolism (PATE), acute myocardial infarction (AMI), and acute cerebral infarction (ACI). Results: FRMs showed the usefulness in diagnosing DIC and VTE and the cutoff values of D-dimer, FDP, and SF for DIC were 7.2–7.8 μg/mL, 10.0 μg/mL, and 9.5 μg/mL, respectively. The cutoff values of D-dimer and FDP for VTE were similar to the 97.5th percentile values of healthy volunteers, while the cutoff value of SF was 6.9 μg/mL. In AMI and ACI, the cutoff values of D-dimer and FDP were lower than the 97.5 percentile values of healthy volunteers. A receiver operating characteristic analysis for all thrombosis cases showed that an adequate cutoff value in only SF among FRMs was higher than the confidence interval of healthy volunteers. Only SF had high sensitivity for thrombosis, as the FDP/SF ratio was markedly low for ACI, AMI and VTE. Conclusions: FRMs, especially D-dimer and FDP, were useful for diagnosing thrombosis with hyperfibrinolysis (e.g., DIC). As SF showed high sensitivity for predominantly thrombotic diseases, including arterial thrombosis, such as ACI and AMI, a high SF value suggests the possibility of an association with thrombosis. Finally, SF is the most useful marker for raising suspicion of an association with thrombosis, especially arterial thrombosis.

## 1. Introduction

Fibrin-related markers (FRMs) include D-dimer, fibrinogen, and fibrin degradation products (FDPs), as well as soluble fibrin (SF). D-dimer is formed by the degradation of polymerized fibrin into dimerized units [1]. SF is the soluble monomeric form of fibrin after fibrinogen is activated by thrombin [2,3]. FDPs include D-dimer, which are involved in both fibrinolysis and fibrinogenolysis. SF may be more specific to thrombosis, as SF has less of an effect on fibrinolysis or fibrinogenolysis [2,3]. The FDP/SF and FDP/D-dimer ratios signify the “fibrin- and fibrinogenolysis/coagulation ratio” and “fibrin- and fibrinogenolysis/fibrinolysis ratio” and can be used to evaluate the balance of fibrinolysis and coagulation or fibrinolytic power [4,5].

Elevation of FRMs are usually observed in patients with disseminated intravascular coagulation (DIC) [6,7] and venous thromboembolism (VTE) [8]. D-dimer is used in many scoring systems for DIC [6,7] and is also used as a marker for the exclusion or prediction of VTE in Europe and North America [8,9,10] and Japan [11,12]. In addition, the utility in predicting postoperative VTE is canceled by anticoagulation therapy [13,14,15], as massive bleeding in patients undergoing major operations is associated with elevated levels of D-dimer [15]. Although various D-dimer kits are now available [16], an adequate cutoff value of each D-dimer for detecting thrombosis has not been determined. Elevated D-dimer levels were associated with a poor prognosis in patients with coronavirus disease 2019 (COVID-19) due to thrombosis [17,18,19,20].

FDPs are frequently used for the diagnosis of DIC in Japan [21,22]. Elevated FDP levels have been reported in patients with aortic aneurysm, trauma, and acute promyelocytic leukemia [23,24,25], suggesting that an elevated FDP indicates hyperfibrinolysis. The FDP/D-dimer ratio also indicates hyperfibrinolysis, and a D-dimer kit with a low FDP/D-dimer ratio strongly reflects hyperfibrinogenolysis [5].

Soluble fibrin (SF) is considered useful for diagnosing thrombosis. Soluble fibrin (SF) is a form of fibrinogen that is activated by thrombin and is considered useful for diagnosing thrombosis [5]. However, adequate cutoff values for each thrombotic disease have not been established using receiver operating characteristic (ROC) analysis. In this study, plasma levels of SF, D-dimer, and FDP were measured in critically ill patients with or without thrombotic diseases, including DIC, venous thromboembolism (VTE), pheriheral artery thromboembolism (PATE), acute myocardial infarction (AMI), and acute cerebral infarction (ACI), to determine adequate cutoff values for each thrombotic disease.

## 2. Materials and Methods

FRMs were examined in 542 patients with thrombosis, including DIC and pre-DIC (135 patients and 181 samples; median age, 78.0 years; 25–75 percentile, 68.5–83.0 years; 53 females and 82 males), VTE (21 patients and 53 samples; median age, 69.5 years; 25–75 percentile, 62.0–82.0 years; 13 females and 8 males), PATE (11 patients and 26 samples; median age, 79.0 years; 25–75 percentile, 65.5–82.8 years; 3 females and 8 males), AMI (80 patients and 80 samples; median age, 75.5 years; 25–75 percentile, 66.5–83.0 years; 24 females and 56 males), and ACI (202 patients and 202 samples median age, 76.0 years; 25–75 percentile, 67.0–83.0 years; 87 females and 115 males) patients, who were managed at Mie Prefectural General Medical Center. FRMs were also measured in unidentified clinical syndrome (UCS; *n* = 98; median age, 56.0 years; 25–75 percentile, 47.0–71.0 years; 51 females and 47 males) and in healthy volunteers *(n* = 98; median age, 22.0 years; 25–75 percentile, 20.0–29.0 years; 59 females and 39 males) as a control group. DIC was diagnosed using the Japanese Ministry of Health Labor and Welfare criteria for DIC [21]; DIC scores of ≥7 points and ≥5 to 7 > points were considered to represent as DIC and pre-DIC, respectively. VTE was diagnosed using elevated plasma D-dimer levels, venous ultrasound, or contrast-enhanced computed tomography (CT). PATE was diagnosed using contrast-enhanced CT or angiography. AMI was diagnosed using elevated troponin levels, coronary angiography, and electrocardiography, and ACI was diagnosed using magnetic resonance imaging or contrast-enhanced CT. Patients with UCS were outpatients with some thrombosis-like symptoms, but they were not diagnosed with thrombosis using imaging findings. Plasma levels of antithrombin, protein C, and protein S were examined in all thrombotic patients who were <50 years of age. Accordingly, there were no cases of congenital thrombophilia in this study.

Blood sampling was performed at admission in all patients with AMI or ACI without anticoagulation therapy at diagnosis without anticoagulation therapy, and one or two days after diagnosis with anticoagulation therapy in patients with DIC, VTE, or PAVE. Patients with VTE or PAVE were treated with heparin, and patients with DIC were treated with antithrombin or recombinant human thrombomodulin. Data from plasma with DIC score ≤ 4 were excluded from DIC/Pre-DIC.

Plasma was prepared by centrifugation of citrated blood at 3000 rpm for 15 min and stored at −80 ℃ before the assay. D-dimer A levels were measured using LIASAUTO D-dimer Neo (Sysmex, Kobe, Japan) with an automatic coagulation analyzer (CS-5100; Sysmex). SF, FDP, D-dimers B, and C levels were measured using Iatro SF II (LSI Medience), LPIA FDP-P (LSI Medience), LPIA-ACE D-Dimer II (LSI Medience, Tokyo, Japan), and LPIA-Genesis (LSI Medience), respectively, with a fully automated blood coagulation analyzer, the STACIA system (LSI Medience) [26]. The D-dimer value was expressed in D-dimer units [27].

This study (2019-K9) was approved by the Human Ethics Review Committee of Mie Prefectural General Medical Center and was carried out in accordance with the principles of the Declaration of Helsinki. Informed consent was obtained from each participant.

### Statistical Analyses

The data in this study are shown as the median (25th–75th percentiles) or median (2.5–97.5 percentile). The significance of differences between two groups was analyzed using the Mann-Whitney *U* test. The cutoff values were analyzed by a ROC analysis; cutoff va1ue-1, the adequate cutoff value, was the value for which the sensitivity and specificity were equal, while cutoff value-2 was the upper confidence interval of healthy volunteers. *p* values of <0.05 were considered to be significant. All statistical analyses were performed using the Stat-Flex software program (version 6; Artec Co Ltd., Osaka, Japan).

## 3. Results

**Figure 1:**
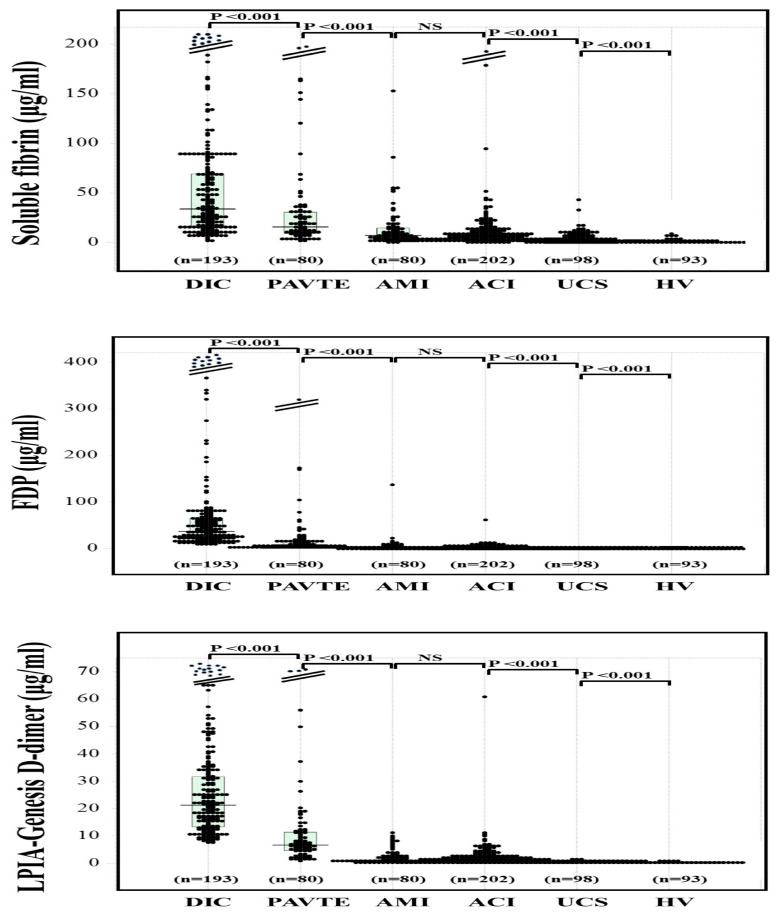
Plasma levels of soluble fibrin (**a**), FDP (**b**) and LPIA-Genesis D-dimer (**c**) in DIC, VTE, PATE, AMI, ACI, UCS, and HV. DIC, disseminated intravascular coagulation; VTE, venous thromboembolism, PATE, peripheral arterial thromboembolism, AMI, acute myocardial infarction; ACI, acute cerebral infarction; UCS, unidentified clinical syndrome; HV, healthy volunteers; FDP, fibrinogen and fibrin degradation product.

**Figure 2:**
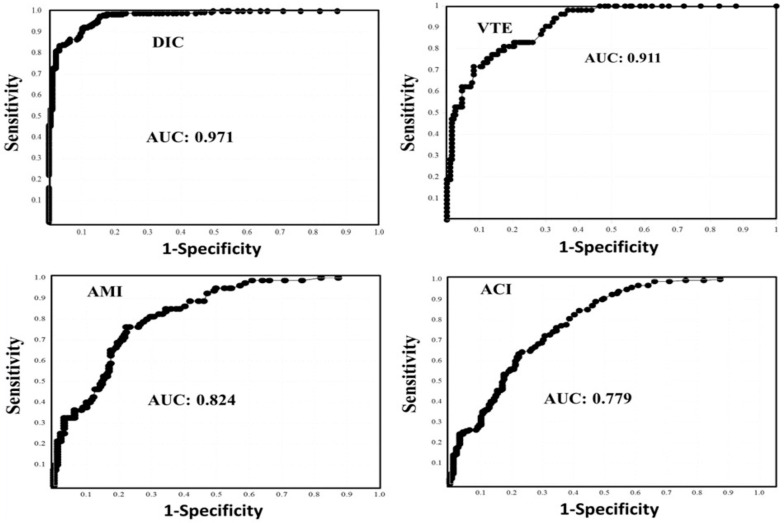
The receiver operating characteristic analysis of soluble fibrin for the diagnosis of thrombosis vs. unidentified clinical syndrome and healthy volunteers. DIC, disseminated intravascular coagulation; VTE, venous thromboembolism, AMI, acute myocardial infarction; ACI, acute cerebral infarction; AUC, area under the curve.

**Figure 3:**
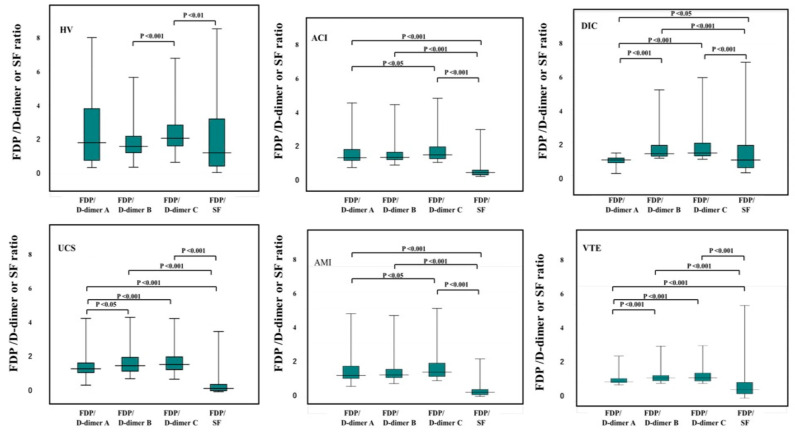
The ratio of FDP/D-dimer or SF in DIC (n = 193), PAVTE (n = 80), AMI (n = 80), ACI (n = 202), UCS (n = 98), and HV (n = 98). DIC, disseminated intravascular coagulation; PAVTE, peripheral arterial and venous thromboembolism, AMI, acute myocardial infarction; ACI, acute cerebral infarction; UCS, unidentified clinical syndrome; HV, healthy volunteers; FDP, fibrinogen and fibrin degradation product; SF, soluble fibrin.

The median values of FDP, SF, and D-dimer A–C in healthy volunteers were similar (0.2–0.5 μg/mL), and the 97.5th percentile values of SF, FDP, and D-dimer A–C were 3.3 μg/mL, 2.1 μg/mL and 0.7–1.1 μg/mL, respectively (Table 1). The plasma levels of SF (Figure 1), FDP (Figure 2), and D-dimer A–C (Figure 3) were significantly higher in patients with thrombotic diseases (e.g., DIC, VTE, PATE, AMI and ACI) than in patients with UCS or healthy volunteers (Table 2). After anticoagulation therapy, plasma leevls of SF, D-dimer, and FDP were significantly decreased in patients with DIC, and data from plasma with DIC score ≤ 4 was excluded from DIC/Pre-DIC. As plasma levels of SF, D-dimer and FDP remained high within two days after diagnosis of VTE or PAVE, and these data were evaluated as VTE or PATE. Although 15 ACI patients and 4 AMI patients were treated with anticoagulant at the diagnosis of ACI and AMI, there was no significant difference between ACI and AMI patients with and without anticoagulant therapy.

In ACI patients, 99 patients were diagnosed with atherosclerotic or lacunar ACI. Plasma levels of FDP and D-dimer A–C were significantly lower in patients with atherosclerotic or lacunar ACI than in those with other ACI. However, there was no significant difference in plasma SF levels between two groups (Table 3). Sixty-three ACI patients were previously treated with anti-thrombotic agents, such as anti-platelet agents, warfarin, and direct oral anticoagulants. There was no significant difference in plasma levels of SF, FDP, and D-dimer A–C between AIC patients treated with and without anti-thrombotic agents (Table 3). 

In the ROC analysis of FRMs for the diagnosis of DIC vs. UCS and healthy volunteers, all FRMs had an area under the curve (AUC) of ≥0.971. In this analysis, the adequate cutoff values of D-dimer A–C, FDP, and SF were 7.8 μg/mL, 7.2 μg/mL, 7.8 μg/mL, 10.0 μg/mL, and 9.5 μg/mL, respectively. All of these cutoff values, which were higher than the normal range, showed high sensitivity and high specificity (Figure 2 and Table 4). In the ROC analysis of FRMs for the diagnosis of VTE vs. UCS and healthy volunteers, all FRMs had an AUC of ≥0.911. The adequate cutoff values of D-dimer A–C determined in this analysis were similar to the 97.5 percentile values of healthy volunteers, while those of SF and FDP were 6.9 μg/mL and 2.4 μg/mL, respectively (Figure 2 and Table 3). In the ROC analysis of FRMs for the diagnosis of AMI vs. UCS and healthy volunteers, all FRMs had an AUC of ≥0.824, and the only adequate cut-off value identified in this analysis was that of SF, which was higher than the 97.5th percentile value of healthy volunteers. Only the 97.5th percentile value of SF in healthy volunteers had high sensitivity for AMI (Figure 2 and Table 4). 

In the ROC analysis of FRMs for the diagnosis of ACI vs. UCS and healthy volunteers, although the AUC was not low, the cutoff values of all FRMs were lower than 97.5th percentile values of healthy volunteers (Table 4). The sensitivity of the 97.5 percentile value in healthy volunteers for the diagnosis ACI was only high for SF (Figure 2).

In the ROC analysis of FRMs for the diagnosis of all thromboses (DIC, VTE, PATE, AMI and ACI vs. UCS and healthy volunteers, although the AUC was high for FDP and D-dimer A–C, those adequate cutoff values were within the confidence interval of healthy volunteers (Table 3). Only SF showed that its adequate cutoff value was higher than the confidence interval of healthy volunteers, although its AUC was not extremely high. 

The FDP/SF ratio in UCS, VTE, AMI, ACI, and DIC was the lowest among the FDP/FRM ratios (Figure 3). The FDP/D-dimer A ratio was similar to 1.0, and the FDP/D-dimer-C ratio was significantly higher than the FDP/D-dimer A ratio in UCS, PAVTE, AMI, ACI, and DIC. The median value of the FDP/SF ratio was 1.4 in healthy volunteers, 1.0 in DIC, 0.6 in VTE, 0.4 in ACI, 0.3 in AMI, and 0.2 in UCS.

## 4. Discussion

The plasma FRM levels increased in patients with thrombotic diseases, especially DIC and VTE. The markedly high AUC values determined in the ROC analysis demonstrated that FRMs, especially D-dimer, are useful for the diagnosis of DIC and VTE [27]. Elevated D-dimer levels are related to the DIC score and associated with poor outcomes [6,28]. However, the overt-DIC diagnostic criteria established by the International Society of Thrombosis and Haemostasis (ISTH) do not include an adequate cutoff value of D-dimer [29], and the scoring system of sepsis-induced coagulopathy dose not include D-dimer [30,31]. There are many D-dimer kits, which have many different cutoff values [16]; thus, the standardization of D-dimer has not been established. Although D-dimer levels varied among D-dimer A–C in this study, the adequate cutoff values for DIC/Pre-DIC using ROC analysis were similar (7.2–7.8 μg/mL), suggesting that the adequate cutoff value for diagnosing DIC may be 7.0 μg/mL in fibrin units or 3.5 μg/mL in fibrinogen units. This cutoff value was in agreement with a previous report [16].

In analysis of ACI, plasma levels of FDP and D-dimers were significantly lower in atherosclerotic or lacunar ACI, suggesting that FDP and D-dimers are not sensitive for artherial thrombosis. However, plasma SFs were not lower in atherosclerotic or lacunar ACI, suggesting that only SF level may be useful for detecting the hyperoagulability in artherial thrombosis, such as ACI or AMI. The absence of significant difference in FDP, D-dimer, or SF levels was observed between ACI patients treated with and without antithrombotic agents as antiplatelet agents, suggesting that antiplatelet treatment may not affect the diagnosis of thrombosis using FDP, D-dimer, or SF.

The adequate cutoff values of D-dimer A–C for VTE using ROC were similar to the 95th percentile of healthy volunteers, indicating that D-dimer values, more than the exclusion value, suggest the possibility of VTE. The adequate cutoff value of SF for VTE was two-fold higher than the 97.5th percentile of healthy volunteers, while the adequate cutoff values of D-dimer A–C for AMI or ACI determined by the ROC analysis were lower than the 97.5th percentile of healthy volunteers, suggesting that these cutoff values are not clinically useful. The plasma levels of D-dimer are reported to be slightly high in patients with ACI, especially cardiogenic ACI [32]. D-dimer may be useful for the diagnosis of cardiogenic ACI. Although the specificity was not high for the diagnosis of ACI or AMI, it showed high sensitivity for the diagnosis of these conditions. That is, elevated SF may suggest the complication of ACI or AMI. Although FRMs have very high sensitivity for thrombosis with hyperfibrinolysis, they have low sensitivity, especially D-dimer and FDP, for thrombosis without hyperfibrinolysis, whereas SF has a relatively high sensitivity for thrombosis without hyperfibrinolysis. 

The FDP/D-dimer ratio was reported to depend on fibrinolysis [33,34], and a D-dimer kit with a high FDP/D-dimer ratio was suggested to be clinically useful [5]. The D-dimer C kit with a high FDP/D-dimer ratio tended to have better results in this study. On the other hand, the FDP/SF ratio was significantly lower than the FDP/D-dimer A–C ratio in VTE, AMI, or ACI, indicating that SF values were higher than FDP or D-dimer A–C values in these diseases. However, the FDP/SF ratio was also high in UCS, suggesting that the specificity of SF is not very high for thrombosis. Therefore, the FDP/SF and FDP/D-dimer ratios were not very useful parameters for diagnosing thrombosis according to a ROC analysis. As SF also showed high sensitivity for all thrombotic diseases that were analyzed in this study, increased levels of SF suggest the possibility of thrombotic complications without hyperfibrinolysis [13].

**Figure 4:**
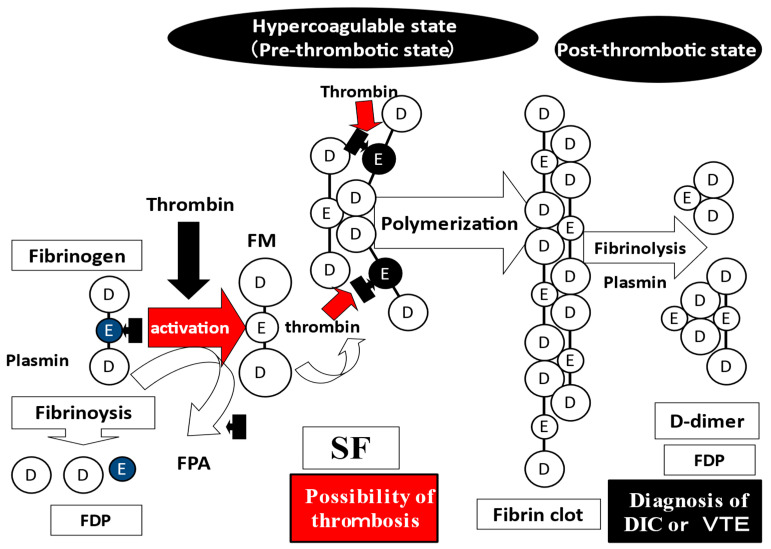
Soluble fibrin. SF, soluble fibrin; FDP, fibrinogen and fibrin degradation product; DIC, disseminated intravascular coagulation; VTE, venous thromboembolism; FPA, fibrinopepetide A.

Although there are a few markers for AMI, such as troponin, that are more useful than SF, there are no useful markers for ACI. FRMs, especially D-dimer and FDP, are not a very useful markers for ACI or AMI without hyperfibrinolysis, but elevated SF levels suggest the possibility of thrombosis, including AMI and ACI. SF consists of an activated fibrin monomer with two fibrinogens, indicating a hypercoagulable state or prethrombotic state. D-dimer is a fibrin degradation product, indicating a post thrombotic state or hyper fibrinolytic state (Figure 4). SF may also be useful for monitoring anticoagulant therapy in patients with thromboses. 

The present study was associated with some limitations. The sample size for VTE and PATE were insufficient for the analysis of the ROC. Furthermore, the age of the patients was not sufficiently matched to that of the healthy volunteers; however, the age was similar to that of patients with UCS. 

## 5. Conclusions

FRMs, including D-dimer, FDP, and SF, are useful for diagnosing thrombosis with hyperfibrinolysis, such as DIC. Only SF has high sensitivity for predominantly thrombotic diseases, such as ACI and AMI, and increased SF suggests the possibility of thrombotic complications without hyperfibrinolysis, such as AMI or ACI. Finally, SF is the most useful marker for suggesting an association with all thrombosis, especially arterial thrombosis. 

## Figures and Tables

**Table 1:** D-dimer, soluble fibrin, and FDP in healthy volunteers.

		Median	2.5–97.5 Percentile
Soluble fibrin	µg/mL	0.3	0.0–3.3
FDP	µg/mL	0.5	0.1–2.1
D-dimer (A)	µg/mL	0.3	0.1–1.0
D-dimer (B)	µg/mL	0.3	0.1–1.1
D-dimer (C)	µg/mL	0.2	0.1–0.7

FDP, fibrinogen and fibrin degradation product.

**Table 2:** D-dimer, soluble fibrin and FDP in DIC, VTE, PATE, AMI, and UCS.

	N	Soluble Fibrin	FDP	D-Dimer (A)	D-Dimer (B)	D-Dimer (C)
		(µg/mL)	(µg/mL)	(µg/mL)	(µg/mL)	(µg/mL)
DIC/Pre-DIC	181	35.3 (16.1–82.2)	36.9 (22.0–64.6)	32.3 (21.2–56.1)	24.5 (16.5–40.0)	22.3 (14.3–35.1)
VTE	53	16.2 (8.7–36.5)	10.2 (6.4–24.3)	9.0 (6.1–18.7)	8.4 (5.8–18.1)	7.5 (5.7–16.8)
PATE	26	19.4 (9.2–27.1)	4.7 (1.8–9.1)	4.9 (2.2–12.3)	4.7 (2.0–9.5)	4.7 (1.8–9.1)
AMI	80	7.1 (3.5–14.4)	1.5 (0.9–3.4)	0.9 (0.5–2.8)	1.1 (0.6–2.9)	1.0 (0.6–2.5)
ACI	202	5.7 (2.3–11.5)	1.6 (1.0–10.7)	1.2 (0.5–2.4)	1.2 (0.7–2.3)	1.2 (0.7–2.4)
UCS	98	3.0 (1.5–7.6)	0.8 (0.6–2.2)	0.5 (0.5–0.5)	0.5 (0.4–0.7)	0.5 (0.3–0.7)
HV	98	0.3 (0.1–0.9)	0.5 (0.4–0.7)	0.3 (0.1–0.4)	0.4 (0.2–0.4)	0.2 (0.1–0.4)
With thrombosis	542	12.4 (5.2–33.3)	6.2 (1.5–27.7)	5.4 (1.1–25.2)	5.3 (1.1–19.6)	5.2 (1.1–17.2)
Without thrombosis	196	1.2 (0.3–3.3)	0.6 (0.4–0.8)	0.5 (0.4–0.5)	0.4 (0.3–0.6)	0.3 (0.2–0.5)

Data are shown as the median (25–75 percentile). FDP, fibrinogen, and fibrin degradation product; DIC, disseminated intravascular coagulation; VTE, venous thromboembolism, PATE, peripheral arterial thromboembolism, AMI, acute myocardial infarction; ACI, acute cerebral infarction; UCS, unidentified clinical syndrome; HV, healthy volunteer.

**Table 3:** Plasma levels of FDP, D-dimer, and soluble fibrin between patients with atherosclerotic or lacuna ACI and other ACI and between ACI patients treated with and without antithrombotic agents.

	Soluble Fibrin (μg/mL)	FDP (μg/mL)	D-Dimer A (μg/mL)	D-Dimer B (μg/mL)	D-Dimer C (μg/mL)
Atherosclerotic or lacuna ACI	4.7 ^NS^ (2.4–11.4)	1.3 ** (0.8–2.5)	1.1 * (0.5–2.0)	1.0 ** (0.6–1.9)	1.0 ** (0.6–1.8)
Other ACI	5.9 ^NS^ (2.3–11.4)	2.1 ** (1.1–3.5)	1.6 * (0.6–3.0)	1.5 ** (0.7–2.9)	1.4 ** (0.7–2.8)
ACI without antithrombotic agents	5.6 ^NS^ (2.2–10.7)	1.7 ^NS^ (1.0–3.1)	1.2 ^NS^ (0.5–2.5)	1.2 ^NS^ (0.6–2.5)	1.1 ^NS^ (0.7–2.4)
ACI with antithrombotic agents	6.5 ^NS^ (2.6–12.7)	1.4 ^NS^ (0.9–2.6)	1.2 ^NS^ (0.5–2.3)	1.1 ^NS^ (0.6–2.5)	1.2 ^NS^ (0.7–2.4)

ACI, acute cerebral infarction; FDP, fibrinogen and fibrin degradation product; D-dimer (A), D-dimer (A), LIASAUTO D-dimer Neo; D-dimer (B), LPIA ACE-D-dimer; D-dimer (C), LPIA genesis D-dimer; NS, not significant; **, *p* < 0.01; *, *p* < 0.05.

**Table 4:** ROC analysis of FDP, D-dimer, and SF for disseminated intravascular coagulation, venous thromboembolism, acute myocardial infarction, acute cerebral infarction, and all thromboses.

	AUC	Cutoff Value-1	Sensitivity	Odds Ratio	Cutoff Value-2	Sensitivity	Specificity	Odds Ratio
Disseminated intravascular coagulation								
SF	0.971	9.5 μg/mL	90.0%	81.9	3.3 μg/mL	97.5%	76.7%	315
FDP	1.000	10.0 μg/mL	99.5%	-	2.1 μg/mL	100%	100%	-
D-dimer A	1.000	7.8 μg/mL	99.5%	-	1.2 μg/mL	100%	100%	-
D-dimer B	1.000	7.2 μg/mL	99.5%	-	1.2 μg/mL	100%	100%	-
D-dimer C	1.000	7.8 μg/mL	99.5%	-	1.2 μg/mL	100%	100%	-
Venous thromboembolism								
SF	0.911	6.5 μg/mL	81.1%	18.5	3.3 μg/mL	83.0%	76.0%	13.9
FDP	0.997	2.0 μg/mL	96.4%	808	2.1 μg/mL	96.2%	97.4%	974
D-dimer A	0.999	1.1 μg/mL	98.1%	2513	1.2 μg/mL	98.1%	98.6%	3796
D-dimer B	0.997	1.3 μg/mL	98.1%	2496	1.2 μg/mL	98.1%	96.4%	1404
D-dimer C	0.999	1.1 μg/mL	98.4%	3345	1.2 μg/mL	98.1%	99.5%	10,140
Acute myocardial infarction								
SF	0.824	3.3 μg/mL	76.3%	10.6	3.3 μg/mL	76.3%	76.3%	10.6
FDP	0.824	0.9 μg/mL	77.5%	11.6	2.1 μg/mL	45.0%	97.4%	25.6
D-dimer A	0.832	0.5 μg/mL	73.9%	10.0	1.2 μg/mL	40%	98.6%	48.7
D-dimer B	0.850	0.6 μg/mL	75.0%	11.3	1.2 μg/mL	47.5%	96.4%	24.4
D-dimer C	0.872	0.5 μg/mL	78.8%	12.3	1.2 μg/mL	43.8%	99.4%	151.7
Acute cerebral infarction								
SF	0.779	2.7 μg/mL	70.3%	5.5	3.3 μg/mL	64.4%	76.7%	6.0
FDP	0.851	0.9 μg/mL	78.2%	12.8	2.1 μg/mL	40.1%	97.4%	25.6
D-dimer A	0.853	0.5 μg/mL	75.2%	11.2	1.2 μg/mL	46.5%	98.6%	63.5
D-dimer B	0.875	0.6 μg/mL	79.8%	15.8	1.2 μg/mL	48.5%	96.4%	25.4
D-dimer C	0.892	0.6 μg/mL	80.2%	17.5	1.2 μg/mL	47.5%	99.4%	176.6
All thrombosis								
SF	0.877	4.0 μg/mL	78.8%	13.6	3.3 μg/mL	81.6%	76.7%	14.6
FDP	0.923	1.0 μg/mL	84.2%	26.2	2.1 μg/mL	69.7%	97.4%	88.0
D-dimer A	0.922	0.5 μg/mL	84.3%	25.4	1.2 μg/mL	71.5%	98.6%	183.4
D-dimer B	0.932	0.7 μg/mL	85.0%	33.6	1.2 μg/mL	73.3%	96.4%	74.3
D-dimer C	0.942	0.7 μg/mL	85.5%	44.2	1.2 μg/mL	72.4%	99.5%	512.4

ROC, receiver operating characteristic; FDP, fibrinogen and fibrin degradation product; SF, soluble fibrin; cutoff value-1, adequate cutoff value determined by ROC analysis; cutoff value-2, upper confidence interval of healthy volunteers. Red color shows the clinically useful cutoff value, which is the upper confidence interval of healthy volunteers. Blue color shows the highest sensitivity with a cutoff value of 2.

## Data Availability

The data presented in this study are available upon request from the corresponding author. The data are not publicly available due to privacy restrictions.
